# Energy and Performance Analysis of Lossless Compression Algorithms for Wireless EMG Sensors

**DOI:** 10.3390/s21155160

**Published:** 2021-07-30

**Authors:** Giorgio Biagetti, Paolo Crippa, Laura Falaschetti, Ali Mansour, Claudio Turchetti

**Affiliations:** 1 DII—Dipartimento di Ingegneria dell’Informazione, Università Politecnica delle Marche, Via Brecce Bianche 12, I-60131 Ancona, Italy; p.crippa@univpm.it (P.C.); l.falaschetti@univpm.it (L.F.); c.turchetti@univpm.it (C.T.); 2 Lab-STICC—Laboratoire des Sciences et Techniques de l’information de la Communication et de la Connaissance, UMR 6285 CNRS, ENSTA Bretagne, 2 Rue F. Verny, 29806 Brest, France; ali.mansour@ensta-bretagne.fr

**Keywords:** EMG, lossless compression, Bluetooth Low Energy, FLAC, entropy coding, wireless sensors, power optimization

## Abstract

Electromyography (EMG) sensors produce a stream of data at rates that can easily saturate a low-energy wireless link such as Bluetooth Low Energy (BLE), especially if more than a few EMG channels are being transmitted simultaneously. Compressing data can thus be seen as a nice feature that could allow both longer battery life and more simultaneous channels at the same time. A lot of research has been done in lossy compression algorithms for EMG data, but being lossy, artifacts are inevitably introduced in the signal. Some artifacts can usually be tolerable for current applications. Nevertheless, for some research purposes and to enable future research on the collected data, that might need to exploit various and currently unforseen features that had been discarded by lossy algorithms, lossless compression of data may be very important, as it guarantees no extra artifacts are introduced on the digitized signal. The present paper aims at demonstrating the effectiveness of such approaches, investigating the performance of several algorithms and their implementation on a real EMG BLE wireless sensor node. It is demonstrated that the required bandwidth can be more than halved, even reduced to 1/4 on an average case, and if the complexity of the compressor is kept low, it also ensures significant power savings.

## 1. Introduction

Electromyography (EMG), i.e., the recording of the electrical activity of muscles by means of suitable electrodes placed on the skin (surface EMG) or inserted into the muscles through needles (intramuscular EMG), is an important tool that is now available to physicians and scientists. It can be exploited to help diagnose neuromuscular disorders and assist in the patient rehabilitation [1], prevent premature birth [2], control prosthetic implants, and more [3]. In its simpler, non-invasive form of surface EMG, it has also been proved to be useful in sports, fitness, and general recreational activities as a means of evaluating muscle fatigue [4,5] and aid automatic diarization [6,7].

Due to the need of recording signals from the muscles while the subject is moving or performing its daily activities, modern EMG sensors are mainly wireless [8,9,10]. For them, minimizing power consumption is crucial, and that can be achieved by carefully designing the analog acquisition chain [11,12], or even by moving the computation to the edge, i.e., on the sensor node itself, which is a hot topic in current research, and can allow significant savings on transmission energy requirements by developing ad-hoc integrated circuits, such as [13]. But for many research purposes, having access to the raw EMG data is still necessary, so it comes at no surprise that in a wireless sensor the possibility of compressing data, to save the limited transmission bandwidth and hopefully also power, is a very desirable property, and many studies have been done on this very topic.

Nevertheless, compressing data often comes with several drawbacks. Many compression algorithms operate on blocks of data, and so introduce considerable latency in the transmission, and also require non-negligible computational power on the sensor that might, or might not, be compensated by the savings in the energy required for transmission. Indeed, with the advent of modern low-power wireless communication technologies such as Bluetooth Low Energy (BLE), the energy required for radio transmission can be optimized through a careful selection of the so-called connection parameters [14], and dedicated scheduling strategies [15]. This, combined with the fact that modern transceivers (like the Nordic Semiconductor nRF52840 used in this study) incorporate high energy-efficient radio modules [16] that only require few milliwatts while active, totally comparable with the CPU power consumption, makes the trade-off between compression effort and energy efficiency non-trivial.

Moreover, the compression algorithms with the highest compression factors are also lossy, which means that they introduce artifacts into the data. These artifacts are generally currently deemed to be within reasonable limits, but that might eventually turn out not to be so, as the scientific knowledge improves and currently unforseen or neglected signal features might one day become important.

This study thus proposes to analyze the effectiveness of lossless compression algorithms to be implemented within wireless EMG sensors, with a particular emphasis on their energy consumption because that is of course the parameter that mostly influences the sensor run time and thus the possible applications of the sensors itself.

After investigating different compression options, we implemented two algorithms onto a wireless EMG sensor and obtained a saving of about 50% in both the energy necessary for processing and transmission and in the used bandwidth, using just a very simple and zero-latency variable-length encoding scheme. More interestingly, using a customized implementation of the free lossless audio codec (FLAC), adapted to real-time streaming of EMG data, we could save about 2/3 of the energy and 60% of the bandwidth while keeping the latency around 1/4 of a second. These results are for the portion of the signal which is less compressible. On average, our FLAC implementation saves over 75% of the original bandwidth.

This paper is organized as follows. Section 2 presents a few relevant related works to set the present study in context. Section 3 describes the different compression algorithms considered in this study and how they have been implemented, measured, and evaluated on a real wireless EMG sensor. Section 4 presents the results obtained by our evaluation, which are then further discussed and analyzed in Section 5. Finally, some conclusions are drawn in Section 6.

## 2. Related Works

Most of the well-known general-purpose lossless compression algorithms are based on variants of the Lempel-Ziv [17] algorithm, which basically scans the data stream for occurrences of patterns present in an evolving dictionary, and then emit indices into that dictionary instead of the matched pattern. The indices are then coded with some space-efficient encoding such as Huffman’s. Many variants exist, according to the details of how the dictionary is updated, searched, and sized, which yield to different trade-offs between compression ratio, memory requirements, and computational cost. Two notable mentions are the LZMA and the bzip2 algorithms. LZMA, which stands for Lempel–Ziv–Markov chain algorithm, unlike the other LZ-based ones, uses a Markov chain to model bit streams, and do not require the data to be byte-aligned to be space efficient. Unfortunately, it is quite computationally expensive, especially for compression. The other, bzip2, on the other hand, does not even use a dictionary, but obtains similarly high compression factors by employing a reversible block sorting algorithm [18], which tends to group similar characters together thus facilitating subsequent entropy coding with just Huffman tables. As for LZMA, its high compression factors are achieved at the expense of a very high computational load.

In general, these algorithms are not optimized for use in signal streaming scenarios, and are included here only to define a baseline reference with which to compare the others. For signal transmission, most of the research has focused on lossy algorithms, as they can achieve very high compression factors. A comparison of some of them can be found in [19], where factors of nearly 10 are achieved with wavelet-based compression, at the cost of adding artifacts that reduce the effective number of bits to between 6 and 8 (our signals start with about 20 effective bits of resolution).

Achieving high compression factors with lossless compressors is much more difficult. In case of the electrocardiographic (ECG) signal, ref. [20] developed an optimized variant of the Lempel-Ziv algorithm that can achieve compression factors of about 8, but the ECG signal intrinsically has lower noise than a surface EMG signal and is much more correlated, hence much more compressible.

Very few attempts have been done to losslessly compress the EMG signal. An off-line comparison of some standard compressors, including FLAC, is reported in [21], but no attempt was made to adapt them to real-time streaming. A compression ratio of about 40% was achieved in [22] for an array of electrodes, using the lossless JPEG compression algorithm that also exploits spatial correlation, unavailable in conventional EMG recordings. On the same paper they claim a 1:10 lossy compression, but with a signal-to-noise ratio only slightly above 20 dB, which equal less than 4 bits of effective resolution.

A much higher lossless compression factor, 3.61, is reported in [2], but for an EMG signal sampled at a very low sample rate of 20 Hz, which excludes most of the higher-frequency, less-predictable components of the EMG signal that can extend at up to 500 Hz.

Our approach can obtain a compression factor of 4 on an 800-Hz sampled surface EMG signal. This was achieved by adapting the FLAC [23] compression algorithm. FLAC, as most lossless compression algorithms for time-domain signals, is based on a common architecture: a first stage predictor tries to estimate and model, with a limited set of parameters, the part of the signal that is somehow regular, as a lossy compressor would do. Then, the error made by this first stage is computed and transmitted as well, after an appropriate entropy coding that needs to be optimized to reduce the required bitrate.

In particular, FLAC adopts a linear predictive coding (LPC) preprocessor for its first stage, which simply tries to estimate the next sample as a linear combination of *n* previous samples, and a variation of the Rice entropy coder [24], known as exponential Golomb, as its second stage. Actually, the Rice encoding is a particular case of the Golomb encoding [25] for a power-of-two encoding parameter $m=2^{r}$. The Golomb encoding itself is a computational efficient and optimal encoding if the probability distribution of the numbers to be encoded follows the geometric distribution. Encoding a number *x* works by computing x/m so that x=m q+p, with *q* being the integer part and *p* the remainder, and then by coding *q* with a unary coding (i.e., *q* bits set to 1 followed by a single bit set to 0) and the remainder *p* of the division using *r* bits in standard binary. Of course, storing large *q* is way less efficient than storing large *p*, so the efficiency of the compression strongly depends on the parameter *r* which sets the trade-off between the magnitudes of *q* and *p*, and that can be optimally computed based on the statistics of the data [26]. To partially overcome the inefficiency of storing large *q*, the exponential-Golomb methods uses a variable length binary encoding method, whereas q+1 is simply written down in binary using the minimum possible number of bits, i.e., $1+\lfloor \log_{2}\left(q+1\right)\rfloor$, and the actual number of bits used (minus 1) is indicated by preceding it with the same number of 0 bits. So, instead of using q+1 bits to store *q*, exponential Golomb uses $1+2\lfloor \log_{2}\left(q+1\right)\rfloor$ bits. For simplicity, the above discussion focused on positive numbers. To store negative numbers, their modulus is actually processed, and the sign bit is appended to the right as the least significant bit, so that their distribution is still centered around 0.

Moreover, to be practically efficient on a time-varying signal, the parameter *r* needs to be adjusted as the statistics of the signal vary. To achieve this, FLAC actually partitions the length of the block it processes into 2^*o*^ segments, where *o* is determined by exploring all values up to a predefined maximum partition order, and a different value of *r* is used (and stored) for each segment. This procedure is particularly useful for large block sizes, but it is quite time and energy consumption, as will be shown next.

## 3. Materials and Methods

The main objective of this work is to investigate the effectiveness of lossless compression algorithms directly applied within wireless sensors nodes, where the power consumption of the CPU doing the compression must be traded with the amount of energy saved by transmitting a smaller amount of data. Finding the optimum trade-off thus requires extensive experimentation, as the already available lossless compression algorithms differ hugely in terms of computational complexity, memory requirements, effective compression ratio (which also, of course, largely depends on the data being compressed) and latency. Latency in particular can be a very fundamental parameter for real-time applications, where the streamed EMG signal needs to be analyzed and interpreted within predefined timing constraints.

Methodologically, we performed a preliminary study on the effectiveness of several standard, well-known, compression algorithms on a typical EMG signal. Afterwards, the two most promising have been implemented within the wireless EMG system [10], so that the power consumption of different algorithms and different settings within such algorithms could accurately be analyzed.

### 3.1. EMG Signal Fundamentals and Properties

**Figure 1:**
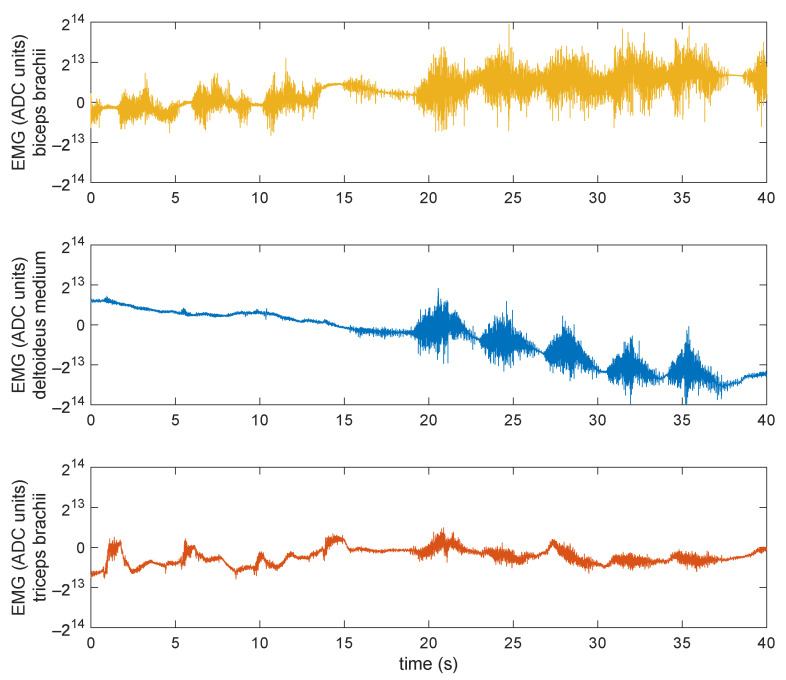
Example of a 3-channel EMG signal recorded from different muscles (*biceps brachii*, *deltoideus medium*, *triceps brachii*) on the upper arm while doing a couple of different exercises as detailed in [10]. The units in the vertical axes are just the output ADC count, after offset removal (the offsets for the three muscles were respectively −12,398, −8725, and +11,999 ADC units). In the *deltoideus medium* the baseline wander still accounts for most of the signal excursion.

A typical EMG signal acquired by our wireless sensor looks like the one reported in Figure 1, where a 40 s window is shown.

**Figure 2:**
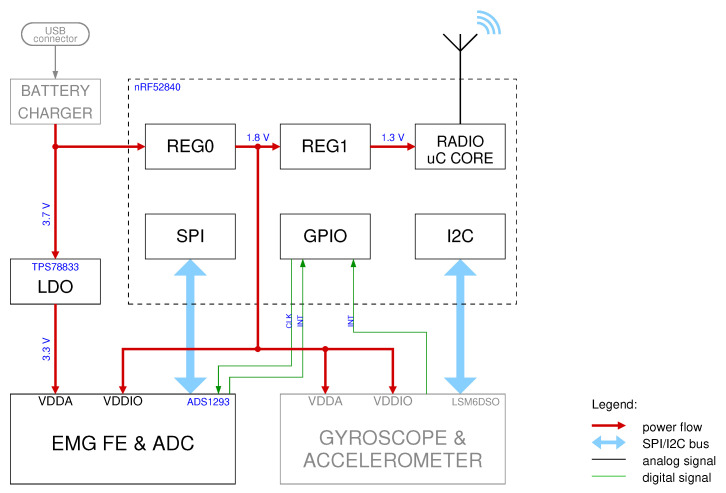
Simplified block diagram of the wireless EMG sensor [10] used for the experiments.

The signal was acquired simultaneously on three different muscles of the upper arm at a sample rate of 800 Hz and transmitted uncompressed over Bluetooth. This alone uses almost half of the available BLE 4.0 bandwidth [27]. The ADC used has an output resolution of 24 bits, with 1 LSB equal to 163.487 nV. Clearly, as will be shown later, its effective number of bits is much lower. According to the chip specifications, it should typically be between 18 and 20 bits depending on sample rate and clock configurations. A simplified block diagram of the sensor is shown in Figure 2, the USB battery charger and the accelerometer/gyroscope blocks have been grayed out as they have not been used in this study.

As can be seen from Figure 1, here the EMG amplitude spans less than 16 bits (about 5 mV analog amplitude) even under high contraction levels. Though the actual level can vary among subjects and recording conditions (electrode types, skin conditions, and body composition playing a very important role), the total signal excursion is typically within 10 mV [3]. What can vary much more than that is the offset (removed from the figure for graphical reasons), especially in the case of DC-coupled electrodes as the ones used here. This will become more problematic if the subject is not still, as the motion artifacts gets superimposed to the signal causing significant baseline wander, hence the need of a large headroom in the ADC chain.

This baseline wander can also be a challenge for some (but not all) compression algorithms so, in the following, we also tried to pre-process the signal by employing a differential encoding, i.e., by transmitting the difference d[n] between a sample x[n] and its predecessor x[n−1]. If the sampling starts at a time n=0, we conventionally assume that x[−1]=0 so that the expression d[n]=x[n]−x[n−1] is always well defined for n≥0, and it allows for perfect signal reconstruction at the receiver since the first transmitted difference is actually just the first signal sample, from which all the following ones can be recovered.

**Figure 3:**
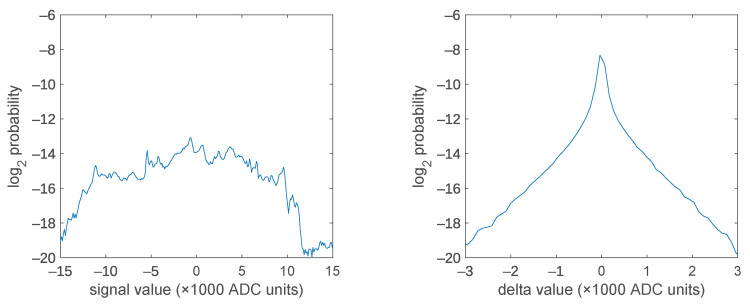
Probability distributions of the (centered) original signals and of their first differences. The entropies associated to these distributions are about 15 bits for the original, and 10 bits for the differential (delta), corresponding to maximum per-sample compression ratios of 61% and 42% respectively. A good compressor should be able to leverage correlation between samples and thus, unless the input is a white noise, should achieve lower rates than the (single) sample entropy.

The effects of such pre-processing on the signal probability distributions were estimated by computing the histograms over a quite long set of measurements, comprising 414,000 samples, measured from different muscles. The results are reported in Figure 3, together with an estimation of the per-sample entropy, defined as $e=-\sum_{i}p_{i}\;\log_{2}p_{i}$, where the index *i* extends over all the possible values of the digitized signal, and $p_{i}$ is the estimated probability of the signal assuming the value *i*. If the data samples were uncorrelated (which they are not), it would results in a maximum theoretical compression ratio c=e/n, where *n* is the original number of bits with which the sample was encoded.

As expected, the differentiation makes values to get highly concentrated around 0, besides having a reduction on their spread by a factor of about 5. This will have an impact on some conventional lossless compressors that otherwise fail to adapt to the correlation between successive samples, but obviously have almost no effect on specialized compressors, like FLAC, that specifically search for the inter-sample correlation.

**Figure 4:**
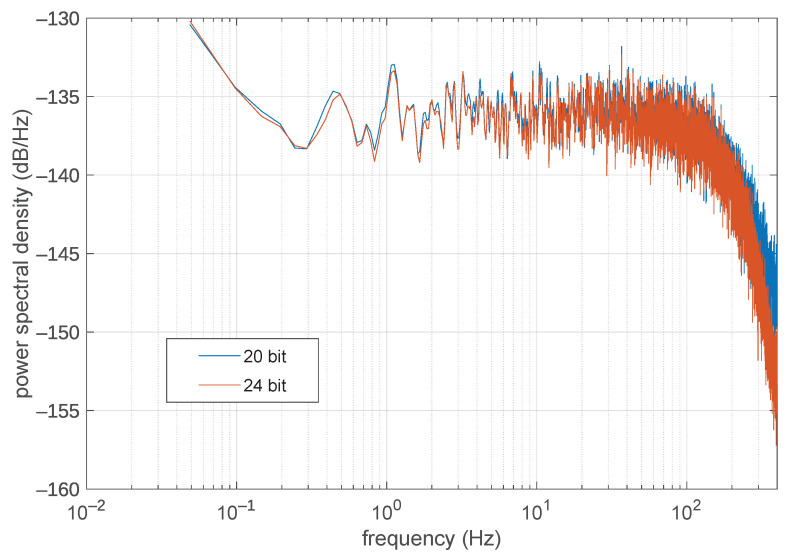
Noise floor vs. number of bits at the output of the ADC.

Another pre-processing that was tested, possibly in addition to differentiation, was the reduction of the ADC output resolution. The sigma-delta ADC employed has indeed a maximum output resolution of 24 bits, but the 4 least significant bits lay below the noise floor of the system, so they can usually be discarded without compromising the signal quality. The effect of this data truncation can be seen in Figure 4, where the power spectral density of the noise floor, as obtained by shorting together the differential inputs of the ADC, is reported.

As it can be seen, the noise floor is essentially the same with both output resolutions. Though in principle word truncation is of course not a lossless operation (because it can reduce or cancel insignificant details of the signal), in this context we deem it to be a property of the ADC output format, as it is customary to do with ADCs having different output resolutions; especially the sigma-delta type, where the number of output bits is often just that of the internal digital filters, and do not directly match their effective analog resolution. With this truncation the estimated per-sample entropy reduces by 4 bits on average, from about 15 bits and 10 bits (original/differentiated signal respectively) to about 11 bits and 6 bits, as expected. Nevertheless, for a more comprehensive evaluation, experiments had still been performed using both the 24 bits per sample data stream and the truncated 20 bits per sample data stream.

### 3.2. Off-Line Compression Ratio Comparison

The same long signal (138,000 samples over 3 simultaneously acquired channels) used to obtain the sample probability density functions was also used to test the effectiveness of five of the most common general-purpose lossless compression algorithms, namely bzip2 (bz2), gzip (gz), LZ4 (lz4), LZMA (xz), and Zstandard (zstd). Since these are general-purpose compressors that do not specifically handle sampled signals, their effectiveness can be affected by factors such as data packing and alignment. Hence, different strategies regarding data alignment had been tested.

In the following, the raw sampled data stream had been encoded in *n*-bit 2-complement binary numbers, with n∈{20,24}, embedded (right justified) into *m* bit words for alignment purposes, with m∈{20,24,32}. Each padding scheme is denoted by the fraction n/m. A total of 5 different combinations had been tested (of course m≥n), whereas the 20 bit per sample signals had been encoded either in packed binary format (20/20), and right aligned to either 24 bits (20/24), or 32 bits (20/32). The 24 bit per sample signals had also been encoded both in packed binary (24/24), and right-aligned into 32 bit words (24/32). Moreover, a simple variable-length encoder (VLDE), described next, and the audio-oriented FLAC compressor have also been added to the tests.

The results are reported in Table 1 for the original signal, and in Table 2 for the differentiated signal. All compressors were set to use their default compression level. The first line of each table (“bin”) represents the size of the binary signal that is being compressed. Of course, data alignment to byte or word boundaries cause an increase in the size of the stream to be compressed, but, as can be seen in the tables, byte alignment actually helped all compressors achieve better performance in the n=20 case. Moving further to word alignment in general did not help, except for LZMA, which often produced (negligibly) better results for m=32.

It must be said that the standard lossless compressors aren’t designed for this use case, so most of them fail to reach even the single sample-entropy compression rate on these signals, as computed from the probability distributions estimated above (which are 42% and 61% for Table 1 for n=20 and n=24, respectively, and 30% and 53% for Table 2), and behave worse than if encoding each sample separately. Only “bz2” and “xz” did better than that, and managed to exploit the inter-sample correlation.

To do so, those compressors use huge block sizes, which are impractical to implement in an embedded system and incompatible with real-time streaming constraints, plus some require disproportionate amounts of memory for encoding, which are simply not available on the sensor nodes. For instance, from the official documentation, at its default “best” setting bzip2 uses 900 kB long blocks, and 7600 kB of memory for compression. LZMA is even more resource-hungry, using 96,256 kB of memory for compression at its default “moderate” setting.

The other three standard compressors (gz, lz4, zstd) use fewer resources and can operate with smaller (but still large for real-time applications) block sizes, but do not offer a satisfactory performance on the EMG signals. Indeed, a simple byte-oriented variable length differential encoding (VLDE) of the data could reach similar performance.

**Figure 5:**
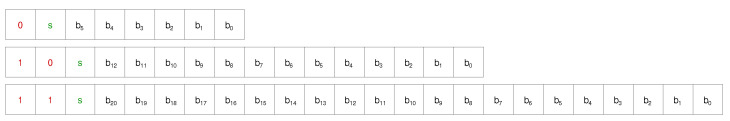
The simple VLDE scheme devised to efficiently send the differentiated EMG signal. Either 8, 16, or 24 bit words are used to store the difference, depending on how many bits are needed to code it in straight binary. “s” is the sign bit, and the fixed red bits allow the discovery of the used word length. 20 significant bits plus sign is exactly the maximum needed to store differences between 20 bit signal samples, so longer words cannot ever be needed.

VLDE is an encoding inspired by that commonly used to encode Unicode text documents, UTF-8. It simply encodes each value independently using 1 byte (if it can be stored in 7 bits), 2 bytes (if it can be stored in 14 bits), or 3 bytes (if it can be stored in 21 bits), using the most significant bits to signal the length of the encoded data, as depicted in Figure 5. Since it operates on a sample-by-sample basis, it has zero encoding latency, which is a very desirable property for real-time streaming, and by only using byte-aligned codewords it allows extremely simple and fast firmware implementations, though it sacrifices some compression efficiency. Indeed, since the 20 bit differentiated signal only has about 6 bits of entropy per sample, most samples (99% actually in our experiment) are encoded in just one byte. Of course, it does not make sense to use VLDE for the non-differentiated signal. Its performance has also been reported in Table 1 for sake of completeness, but, obviously, it turned out to be largely ineffective as data is not centered around 0. On differentiated data (Table 2), instead, it beat most general-purpose compressors.

On the other hand, a compression algorithm born for lossless audio compression behaved much better on the EMG signals as well. It does not have data alignment problems as it explicitly uses a sampled signal as input, and can be tuned to use arbitrarily long (or short) block sizes. Its effectiveness is also reported at the bottom of Table 1 and Table 2 (the reference implementation used for the tests does not allow m=32), and is essentially insensitive to original word size and signal differentiation, being a perfect candidate for embedded usage.

**Figure 6:**
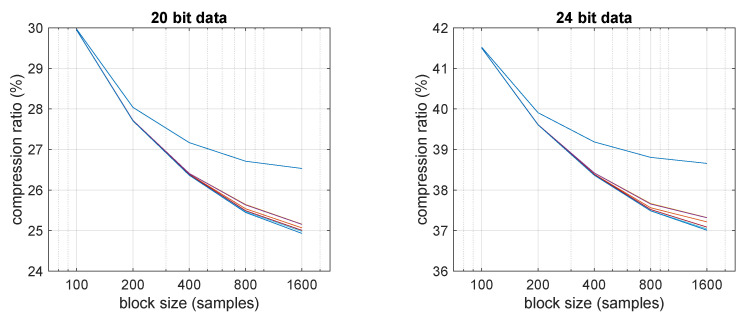
Compression efficiency of FLAC versus block size for different compression levels, from 0 (highest curve) to 7 (lowest curve). Except for very long block sizes, compression levels above 1 have no practical effect on compression ratio. At a sample frequency of 800 Hz, these block sizes translate to latencies from 1/8 s to 2 s, which are still usable for real-time monitoring.

To further investigate how its performance is affected by block size, which basically defines the latency of the system, Figure 6 reports an analysis of the compression ratio versus block size for different compression levels (detailed in the next subsection).

As can be seen, different latency-compression trade-offs are possible with FLAC, and it still outperforms all the general-purpose compressors even at the shortest block size. Based on all the above considerations, it can be stated that the only two compressors, among those tested, that can make sense in an embedded wireless EMG sensors are FLAC and VLDE. In the rest of the paper, we discuss implementation details and power consumption analysis of these two.

### 3.3. FLAC Encoder Implementation on the Sensor Node Firmware

Implementing the FLAC encoder on the sensor node for real-time streaming required some adjustments to the reference implementation, and a completely redesigned buffering strategy. Indeed, although FLAC compresses each channel independently (unless in joint-stereo mode, which is only meaningful for audio and has thus been disabled), the reference implementation takes in interleaved buffers as they are commonly produced by sound cards. These intermediate buffers are not useful in an embedded application and have therefore been removed, and the de-interleaving of the data coming from the ADC was performed simultaneously to data scaling and directly into the buffers used for compression.

Moreover, since the reference implementation needs to be portable across a large variety of systems, and on some of them also implements code optimization using single-instruction multiple-data (SIMD) instructions, which may require very large memory alignments, the FLAC encoder waits to fill the buffers with some look-ahead samples from the next block before starting compression, to ensure no uninitialized data is ever read by SIMD instructions. This unfortunately leads to an increase in latency, up to another full data block in the worst case scenario, which we deemed incompatible with the present need of real-time streaming. After all, the 32 bit CPU used in the sensor node never requires more than 32 bit word alignments, and all the math in the FLAC library is done in 32 bit, and there are no SIMD instructions large enough on the microcontroller to possibly leverage them.

Due to these considerations, the buffering scheme of the reference library was rewritten from scratch. Data acquired by the ADC is quickly moved without any processing into a temporary queue from within the interrupt service routine itself, waiting for the main thread to be free. When the main thread is free, the data into the queue is properly scaled and transferred into the compression buffers, one for each EMG channel, which operate as ping-pong buffers, whereas one is being filled while the compressor operates on the other. When a buffer is filled with the proper block size number of samples, the compressor is invoked on that buffer and compressed data is queued for transmission over BLE.

The BLE firmware can actually queue only a limited number of notifications for transmission. So, in order to avoid congestion and unbounded delays in case of radio interferences, a separate queue is used for output packets, and notifications are only sent when the BLE queue is below a certain threshold, set to limit the number of transmissions to four packets per connection event (uncompressed data only requires three, so there is ample margin).

Besides buffering, the compression presets had also been altered, as some of the default ones made little sense in case of non-audio material. Essentially, each FLAC compression level defines the maximum order used for linear prediction of the signal (LPC order), and for entropy coding of the prediction residual (partition order). Moreover, since the signal is windowed before being analyzed, different apodization functions can be selected for this windowing, and the one yielding the best result is used.

Lower levels always use a standard Tukey window, while higher levels also try some modifications on that, as detailed in Table 3.

As far as the output format, the FLAC standard specifies several checksums to ensure data integrity: an MD5 checksum of all the original uncompressed data should go into the main header, then there are 8 bit CRC to protect each frame header and 16 bit CRC to protect each frame of compressed data. The main MD5 checksum is only meaningful for off-line use, and so it has been disabled. The other per-frame headers and checksums are also redundant in case of a BLE transmission, which uses its own packetization and checksumming strategies. They amount to between 11 and 13 bytes per frame (they contain a variable-length encoded frame counter) and could easily be removed to slightly improve compression ratio (between 0.6% and 0.8% for a 200 samples frame). Nevertheless, we chose to keep them, as they facilitate stream synchronization recovery in case of packet loss and the gain attainable from their removal would be minimal.

### 3.4. Baseline Power Consumption Analysis

Before evaluating the performance of the different compression algorithms, an analysis of the power consumption of the basic sensor node is performed, so that the overall power consumption can be better explained in terms of the requirements of the different subsystems.

All the measures have been carried out by supplying the sensor from its battery terminals, with the nominal 3.7 V battery voltage, using a custom-made source-meter unit we specifically built for this purpose, which is able to supply a configurable voltage while simultaneously sampling the output current.

**Figure 7:**
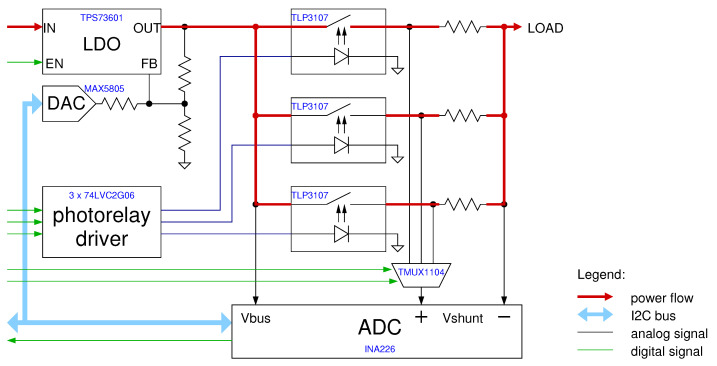
Block diagram of the custom-made source-meter unit. The photorelay driver contains pulse-shaping circuitry to drive the relays with minimum turn-on and turn-off times, allowing make-before-break operation while switching from one sensing resistor to another. The DAC-controlled LDO allows selection of output voltage from 1 V to 5 V in 1 mV increments. The whole system is software controlled by a Raspberry Pi (not shown) that drives the digital signals that control the photorelays and the multiplexers through some GPIO pins, plus acquire the data through its I2C bus.

The current sensing is done through an instrumentation amplifier (Texas Instruments INA226) specifically dedicated for high-side current sensing applications, coupled with three different sensing resistors of 0.1 Ω, 1.0 Ω, and 10 Ω, that can be switched in under software control. These allow the system to have a very large sensing range (of 500 mA, 50 mA, and 5 mA respectively), which is quite necessary for BLE applications given the very low amounts of current drawn during idle periods, and the relatively high peaks during wakeup and radio activity. The resistors are switched by means of fast solid-state relays (Toshiba TLP3107) with custom-designed drivers that guarantee make-before-break operation in less than 0.5 ms for fast range change. The relays have a low, 30 mΩ, on-resistance, which is nevertheless excluded from the sensing chain by virtue of an analog multiplexer (Texas Instruments TMUX1104) that connects only the selected sensing resistor to the amplifier. A DAC-controlled low-dropout (LDO) regulator (Maxim MAX5805 and Texas Instruments TPS73601) completes the power sourcing chain, as illustrated in Figure 7.

**Figure 8:**
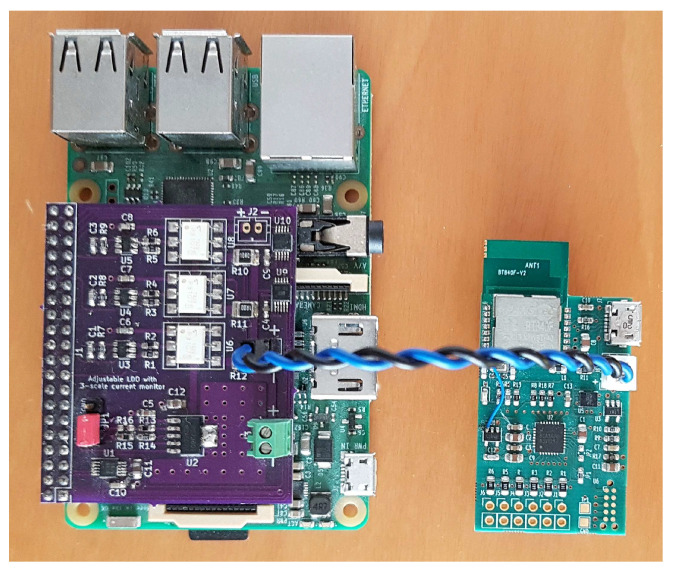
Photograph of the whole system, showing the custom-made source-meter unit (purple board attached on top of the Raspberry Pi, left) powering the EMG wireless sensor board (right) through its battery connector.

Current measurement can be performed at up to a 3 kHz sampling frequency, and sampling times are accurately timestamped at microsecond resolution against absolute time as recorded by a clock servoed to a local time server, so that current measurements can be accurately related to BLE events as recorded from the receiving PC, as shown in [10]. A picture of the whole measurement setup is shown in Figure 8.

To perform power consumption measurements, the BLE link was configured with the minimum allowable connection interval, 7.5 ms, as done in the aforementioned paper so as to allow low-latency streaming of uncompressed data. The BLE link latency was set to 32 (corresponding to 247.5 ms) to conserve power when there is no data to be transmitted (the BLE latency specifies the number of connection events that the node can choose to skip if it does not have any pending notification to be sent, it does not affect the signal latency while transmitting).

**Figure 9:**
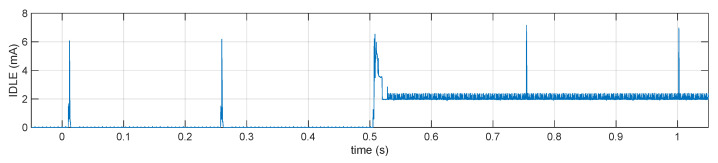
Current consumption of the EMG sensor node in “idle” conditions: for first half second the system was connected over BLE but otherwise idle, the spikes corresponding to radio activity every 247.5 ms. On the third connection event the sensor received the command to turn on the ADC subsystem, the subsequent lower-amplitude spikes are the CPU activity resulting from the ADC interrupts at 800 Hz.

A measurement of the current in idle conditions is shown in Figure 9. When the system is connected over BLE but otherwise idle (i.e., during the first half second shown in the figure), it only draws 430 μA on average from the battery. After 0.5 s the system was commanded to turn on the analog portion including the ADC subsystem. This alone accounts for 2.07 mA of average current consumption, part of which is due to the analog parts themselves and, unfortunately, most is due to the clocking of the ADC, the CPU being mostly idle. This consumption is quite high because at the design stage it was decided to derive the ADC clock (409.6 kHz) from the CPU clock (64 MHz crystal oscillator) using a hardware fractional divider inside the microcontroller. This allowed tight synchronization between the CPU timers and the ADC sampling period, but the fractional divider inside the chip turned out to be the single most energy-consuming subsystem. Replacing it with an external MEMS-based oscillator is expected to at least halve this baseline power consumption.

In the rest of the paper, when comparing the effectiveness of different compression algorithms, this baseline consumption must be taken into account as it represents a hardware-imposed lower limit that no algorithm or software optimization can affect.

## 4. Results

To evaluate the performance of different compression algorithms and formats, some of these have been implemented in the firmware of the EMG sensor node so as to be able to experimentally determine their effective power consumption. To obtain reproducible results, while all the sensor subsystems had been left operational, data from the ADC was discarded and replaced on-the-fly with a pre-stored sequence, which gets looped. This also allowed us to verify that, once decompressed, the received data was actually identical to data before compression, so as to validate our firmware code.

The pre-stored sequence is 256 samples long, longer than the compression block size employed, so as to avoid repeatedly compressing always the same sequence. It was selected to contain a muscle contraction, since it was experimentally determined that those are the signal sections which are less compressible, besides being the ones of greatest interest. The compression ratios shown below are thus worse than those for the whole signal, and can be interpreted as a sort of worst-case scenario for the firmware implementation.

### 4.1. Compression Power Consumption Measures

The BLE standard is based on a mandatory 1 Mb/s physical layer (PHY) radio, and an optional 2 Mb/s PHY to guarantee improved throughput. It also has the possibility of using coded PHYs that add redundancy to the data to augment transmission range but at much lower data rates, as they actually use the 1 Mb/s PHY but at 1/2 or 1/8 data rate to make room for forward error correction.

In this context, we focused only in the non-coded PHYs, as otherwise the bandwidth would not be enough for the uncompressed data. Experiments were repeated using both the 1 Mb/s (1M) and the 2 Mb/s PHY (2M), because the two transceiver data rates obviously require different amount of energy to transmit the same amount of data, hence the best trade-off between compression ratio and computational effort could in principle be different.

Table 4 reports the attained compression ratios and the corresponding average current consumption. The latency specified for the RAW and VLDE cases are just that necessary to fill a notification packet with 18 bytes of payload, so two 3×24 bits samples for RAW (3 EMG channels), and at most six 3×8 bits coded deltas for VLDE. The latency reported for FLAC is just the encoding latency and it equals the block size, the transmission latency was not taken into consideration but for FLAC it amounts to about another half of the block size, as it will be shown later.

The currents reported are the differences with respect to the baseline, as described in the previous section. With such a short block size, several FLAC compression levels produced identical results (and identical compressed streams), so the corresponding lines have been coalesced. The 20 bit RAW format had not been implemented since packing data in a non-byte-aligned structure would require more complex code than VLDE, but without the compression benefits, and so it is quite useless.

As it can easily be seen, and could also be expected, FLAC at its lowest compression setting, level 0, is the clear winner in terms of energy consumption, if the latency can be tolerated. At 24 bit, it halves both the data rate and the energy consumption, and behaves even better at 20 bit. VLDE also performs remarkably well, almost on a par with FLAC at level 1 for power consumption, because it of course has a negligible computational overhead and the energy savings are basically all due to the reduced transmission requirements. It can hence be considered a very appealing choice when low latencies are needed. Higher FLAC levels, on the other hand, require significantly more computational resources for negligible improvements in compression ratio, and are hence of limited interest.

As far as moving from 24 bits to 20 bits samples, again, as the background noise is supposed to be almost white (and it appears to be so, at least in the range from 0.1 Hz to 100 Hz, as show in Figure 4), and white noise is uncompressible, one can expect the 24 bit compressed data stream to require on average at least 4 more bits per sample than the 20 bit compressed data stream. This can indeed be easily seen in Table 4, for instance for the FLAC compressor. At level 0, the 24 bit streams are compressed at 50%, yielding on average 12 bit per sample, while a 20 bit stream is compressed at 40%, yiedling on average just 8 bit per sample, with the 4 bit per sample difference due to the non-compressible white noise present in the “higher-resolution” stream.

### 4.2. CPU Usage Analysis

To further investigate the performance of some of the compression algorithms, a high-time resolution analysis of the current has also been performed, so that individual contributions to the overall power could be better highlighted.

**Figure 10:**
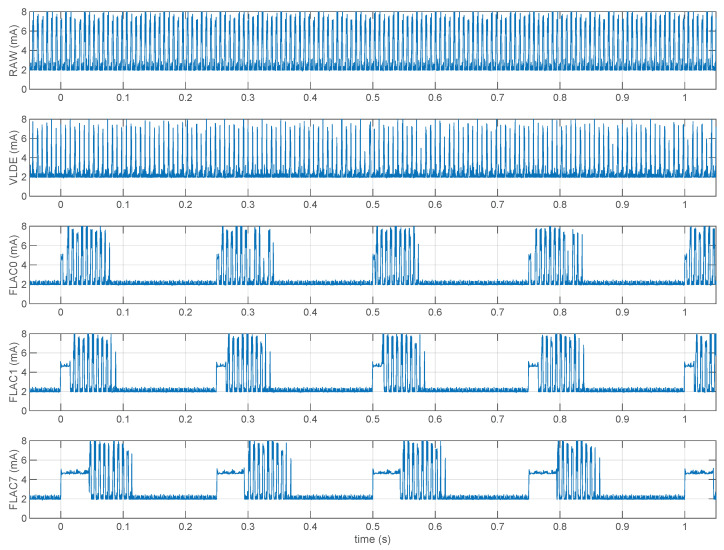
Current consumption of different compression algorithms. The top strip actually represent uncompressed (raw) data streaming. On average, 3 packets per connection event are transmitted. The other strips are for 20 bit per sample compressors, starting from VLDE. It has the same connection event density as the raw data, but it only needs to transmit 1 or 2 packets per connection event (1.5 on average since it compresses about 50%). Next we have FLAC at various levels: after an initial CPU time due to block compression, the software was set to transmit up to 4 packets per connection event, so there is ample radio idle time between bursts. Since the block size was set at 200 and the sample rate is 800 Hz, we have one burst every 250 ms.

Figure 10 shows traces of the node power supply current as obtained with our programmable source meter. From top to bottom, they refer to uncompressed RAW data streaming, to VLDE encoded 20 bit data, and to FLAC compressed 20 bit data, at compression levels 0 (lowest), 1, and 7 (highest).

As can be seen, RAW and VLDE transmit packets at every connection event (we used a 7.5 ms connection interval), though of course VLDE transmits less packets per event. VLDE code is trivial and negligible with respect to all the other code the microcontroller runs e.g., in the ADC interrupt service routine, so its execution cannot be seen on the trace.

On the other hand, FLAC can have a significant impact on CPU utilization. It can clearly be seen as the “block” of current at about 5 mA (3 mA above baseline) starting at multiples of 0.25 s. At level 0 the energy required for compression is almost negligible with respect to that required for transmission, but it is not so at higher levels. Indeed, it is also worth noting that at a 200-samples block size, FLAC levels from 1 to 7 all yield essentially the same compression ratio (Figure 6), but require progressively higher computational resources, which are thus not justified.

From the traces, it is possible to estimate the CPU time required for compression, which are 3.4 ms, 15.5 ms, and 43.8 ms respectively for FLAC levels 0, 1, and 7, corresponding to 1.36%, 6.20%, and 17.52% CPU utilization ratios.

### 4.3. Distribution of Compression Ratios

As mentioned at the beginning of this section, all the experiments that involved the hardware sensor were carried out with a pre-stored sequence of previously-recorded ADC data, so as to ensure repeatability and correctness. Now that the optimal settings for the FLAC compressor have been identified, it can be interesting to see how it performs on a longer and more varied signal.

**Figure 11:**
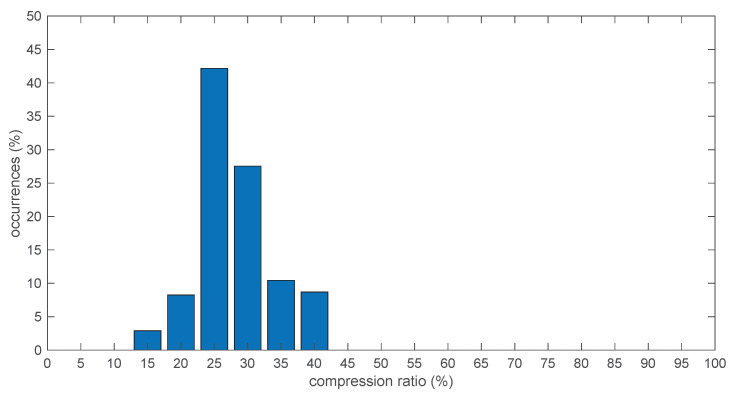
Histogram of the compression ratios achieved by FLAC level 0 over the whole available signal. 20 bits per sample setting, 200 sample block size.

To this end, Figure 11 reports the percentage of frames that were able to achieve a certain compression ratio, computed over the whole signal initially used for the off-line tests, comprising 690 frames, each with 200 samples on 3 channels at 20 bits per sample. From Figure 6, at level 0 FLAC achieved on average a 28% compression ratio, and from Table 4 a “worst-case” compression ratio of 40%. This is indeed confirmed by the histogram shown in Figure 11, from which it can be verified that the selected portion of signal for the in-sensor tests actually corresponded to the worst case, while the vast majority of frames compressed much better at around 25%.

**Figure 12:**
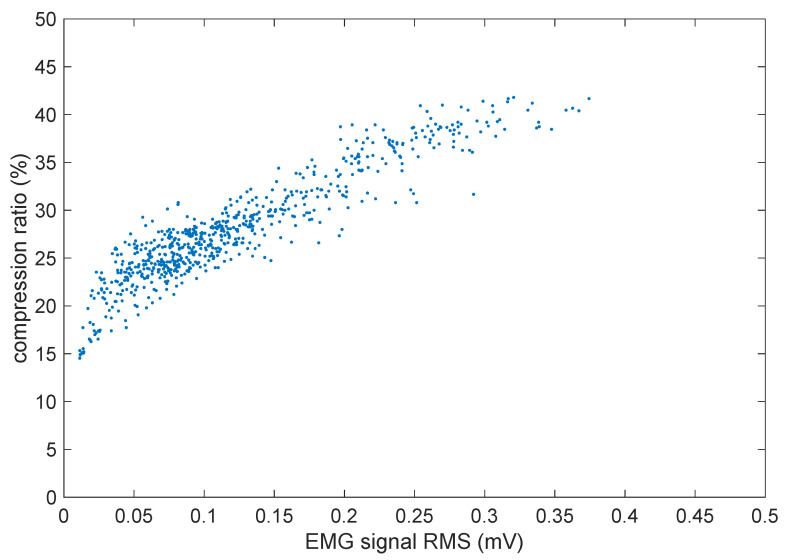
Scatter plot of the compression ratio vs. EMG signal strength (average RMS value of the three channels, as FLAC compresses them together into a single frame).

The compression ratio achieved over each frame is of course strongly dependent on the amount of muscle activity present during that frame. To better appreciate that, Figure 12 shows a scatter plot of the per-frame compression ratio vs. the average root mean square (RMS) value of the three EMG signal segments during the same frame.

As could be expected, there is a direct correlation in that stronger muscle contractions (and hence higher EMG signal levels) lead to less compressible signals, as obviously there is more information to be carried in the signal.

## 5. Discussion

From the above results, it can be stated that FLAC at its lowest compression settings represents a very good compromise for real-time streaming of EMG data. With a latency of a fraction of a second, it allows substantial energy savings, due to the reduced bandwidth usage and almost negligible CPU time required for compression. From Figure 12, it can be seen that the bandwidth is actually more then halved in the worst case, but it is even reduced to a mere 15% for the portions of the signal that have the lowest levels, and those correspond to muscles at rest, which can actually represent the majority of the time in continuous-monitoring applications where the sensor should be worn all day long.

Of course, being a lossless system, the actual compression achieved cannot be guaranteed, as it depends on the particular signal and most importantly on the amount of noise, usually uncompressible, that it contains. It is thus important when designing a complete monitoring system that resources, in terms of bandwidth allocation and buffer sizes for retransmission queues etc., are properly allocated so that the whole wireless network does not collapse when hard-to-compress frames arrive.

To this end, statistical analyses such as those reported in Figure 11 can be very useful to tweak the parameters of the BLE communication layer, but this layer is outside the scope of the present paper and its optimization to carry compressed data may well be the subject of future research.

This study also demonstrated that standard, general-purpose data compressors do not work well for the EMG signal, achieving only modest compression ratios. This is also confirmed by a survey of the literature. Although few studies have been made for lossless EMG signal compression, Table 5 summarizes the most relevant and pertinent results published by those studies.

Starting from the preliminary work [21], where only off-line scenarios have been considered and tests were performed just using utilities commonly available on personal computers, it was apparent that the simplest LZ-based compressors like GZIP performed poorly, and that more advanced systems like BZIP2 and LZMA or specialized ones like FLAC worked better and produced equivalent results in terms of compression. Of course, neither BZIP2 nor LZMA are implementable on a resource-constrained embedded system due to their very large memory footprints. That work also considered the case of resampled (upsampled) and synthetic EMG signals, but those results are not comparable and so have not been reported in the table.

Similar compression results have been achieved in [22], though they actually focused mainly on EMG signals recorded through a two-dimensional array of electrodes. Due to the two-dimensional nature of the data, specialized algorithms such as lossless JPEG could be effectively applied, but again the results are not comparable with our case. They also report the results of a baseline compression made using ZIP, the range of compression ratios correspond to a range of contraction levels that had been tested. The compression ratio might seem slightly worse than others’, but the starting signal resolution was the lowest for that study, at just 12 bits, so it could be expected.

More interesting results have been reported in [2]. Starting from 32 bits better compression ratios could be expected, and are indeed achieved. The authors of [2] propose their own lossless compressions strategy called EUMTS, which is a variable-length encoding somewhat similar to VLDE and that can encode most of the EMG signal deltas in just on byte, thus with a theoretical maximum compression efficiency of 8/32 = 25%. The achieved 28% is thus very good (our result, 42%, might seem much worse, but we start from 20 bits and hence the maximum theoretical ratio is 8/20 = 40%, so the effectiveness is quite similar). Though, it must also be said that the employed sample rate, just 20 Hz, seems a little low to capture the details of the EMG signal and might have contributed to reduce the amount of noise the compressor had to deal with, hence the relatively good results obtained with the baseline LZW and Huffman compressors.

It must be said that, besides our own work, EUMTS was the only one that was implemented in a real-time setting, and that to the best of our knowledge there are no studies of the energy consumption of lossless compression algorithms implemented in a real EMG sensor.

## 6. Conclusions

As wearable devices for continuous monitoring become more widespread, the need to increase battery life of portable sensors is ever growing. Data compression can obviously help tackle the energy consumption problem, but the optimal trade-off between compression ratio and energy reduction is far from obvious as many factors come into play, from compressor algorithmic complexity, going through suitability of implementation into an embedded systems, to the ratio between the energy footprint of computation versus transmission for the specific system being optimized.

In this work, we analyzed in detail both the compression efficiency and the energy consumption of some lossless algorithms suitable for the wireless transmission of the EMG signal. It turns out that a modest compression level, as provided by FLAC at level 0, achieves the maximum energy savings, shaving off up to 75% of the energy required for data processing and transmission. It also reduces the required bandwidth by 60% on the least compressible portions of the EMG signals (strong muscle contractions) and by a factor of 4 on average. The completely redesigned buffering strategy also allowed the reduction of the latency compared to the original reference implementation.

The cost of the higher compression settings, mainly due to introducing higher LPC orders with the need of estimating its coefficients, is not compensated by the modest gains in compression ratio. Indeed, even a modest-performing simple VLDE encoding provides very good energy metrics because, even if it does not compress much, it consumes a negligible amount of energy to do so. On systems employing modern, low-power communications protocols like BLE, it is thus extremely important to take into consideration the cost of computation, even if it means sacrificing a little the compression performance.

## Figures and Tables

**Table 1:** Off-line data compression ratio (percentage) for different compression algorithms and bit depths, applied to the original (non-differentiated) signal. Columns represent different data size/alignment options.

Compression Format	Data Size/Alignment				
	20/20	20/24	20/32	24/24	24/32
bin	100.00	120.00	160.00	100.00	133.33
bz2	35.27	32.54	32.83	46.75	47.53
gz	53.06	49.12	54.16	64.57	70.53
lz4	83.43	74.38	76.45	90.79	102.22
xz	41.34	35.98	35.27	46.04	46.74
zstd	58.69	50.11	53.65	65.79	68.91
VLDE	—	—	78.23	—	90.17
FLAC	25.53	25.62	—	37.59	—

**Table 2:** Off-line data compression ratio for different compression algorithms and bit depths, applied to the differentiated signal. Columns represent different data size/alignment options.

Compression Format	Data Size/Alignment				
	20/20	20/24	20/32	24/24	24/32
bin	100.00	120.00	160.00	100.00	133.33
bz2	29.25	28.01	28.02	41.37	41.38
gz	42.97	40.21	44.80	54.65	60.82
lz4	69.67	66.34	71.08	78.88	82.52
xz	34.77	30.79	29.38	44.87	43.21
zstd	43.12	41.10	45.75	54.34	58.25
VLDE	—	—	42.12	—	52.45
FLAC	25.12	25.19	—	37.17	—

**Table 3:** Parameters corresponding to the various FLAC compression levels as used in this work. They differ slightly from the reference implementation due to the embedded system restrictions and to the fact that we use a 3-channel setup, thus rendering “tricks” such as joint-stereo inapplicable.

FLAC Level	Max LPC Order	Max Residual Partition Order	Apodization Functions
0	0	3	tukey
1	6	4	tukey
2	8	4	tukey
3	8	5	tukey
4	8	5	tukey; partial_tukey
5	8	6	tukey; partial_tukey
6	12	6	tukey; partial_tukey
7	12	6	tukey; partial_tukey; punchout_tukey

**Table 4:** Data compression ratio and processing/transmitting sensor current consumption for different compression formats and different BLE PHY speeds. The baseline current consumption for the analog parts and clocking is 2.07 mA and 2.06 mA for the 1M and 2M PHYs, respectively.

Compression		24 Bit Data			20 Bit Data		
Format	Latency	Ratio	*I* @ 1M	*I* @ 2M	Ratio	*I* @ 1M	*I* @ 2M
	[Samples]	[%]	[mA]	[mA]	[%]	[mA]	[mA]
RAW	2	100	1.34	1.05	100	—	—
VLDE	6	64.497	0.89	0.71	50.418	0.61	0.50
FLAC 0	200	50.060	0.68	0.54	40.047	0.46	0.37
FLAC 1	200	49.289	0.83	0.68	39.167	0.58	0.48
FLAC 2-3	200	49.289	0.87	0.73	39.167	0.61	0.52
FLAC 4-5	200	49.284	0.98	0.84	39.160	0.70	0.60
FLAC 6	200	49.284	1.07	0.93	39.162	0.77	0.67
FLAC 7	200	49.280	1.25	1.10	39.153	0.89	0.80

**Table 5:** Performance comparison with other works.

Ref.	Real Time	Employed Algorithm(s)	EMG Signal Source(s)	Data Rate [Hz] × [Bits]	Compression Ratio
[21]	no	GZIP	*masseter*	1000 × 16	∼75%
[21]	no	BZIP2, LZMA, FLAC	*masseter*	1000 × 16	∼62%
[22]	no	ZIP	*upper trapezius*	1000 × 12	80–85%
[22]	no	ZIP	*medial gastrocnemius*	1000 × 12	62–75%
[2]	yes	EUMTS	uterine EMG	20 × 32	28%
[2]	no	LZW, Huffman	uterine EMG	20 × 32	53%
this work	yes	FLAC	upper arm muscles	800 × 20	28%
this work	yes	VLDE	upper arm muscles	800 × 20	42%

## Data Availability

Not applicable.
